# Cardiovascular disease risk in offspring of polycystic ovary syndrome

**DOI:** 10.3389/fendo.2022.977819

**Published:** 2022-11-30

**Authors:** Noha M. Shawky

**Affiliations:** ^1^ Department of Cell and Molecular Biology, University of Mississippi Medical Center, Jackson, MS, United States; ^2^ Women’s Health Research Center, Mississippi Center of Excellence in Perinatal Research, University of Mississippi Medical Center, Jackson, MS, United States

**Keywords:** cardiovascular disease, hypertension, polycystic ovary syndrome, offspring, sex differences

## Abstract

Polycystic ovary syndrome (PCOS) is the most common endocrine disorder affecting women at reproductive age. PCOS diagnosis (Rotterdam criteria) is based on the presence of two out of three criteria; clinical and/or biochemical hyperandrogenism, oligo- or an-ovulation and polycystic ovaries. PCOS women suffer from a constellation of reproductive and metabolic abnormalities including obesity and insulin resistance. PCOS women also have increased blood pressure and increased risk of cardiovascular diseases (CVD). *In-utero*, offspring of PCOS women are exposed to altered maternal hormonal environment and maternal obesity (for most of PCOS women). Offspring of PCOS women could also be subject to genetic susceptibility, the transgenerational transmission of some of the PCOS traits or epigenetic changes. Offspring of PCOS women are commonly reported to have an abnormal birth weight, which is also a risk factor for developing CVD and hypertension later in life. Although studies have focused on the growth pattern, reproductive and metabolic health of children of PCOS women, very limited number of studies have addressed the risk of hypertension and CVD in those offspring particularly as they age. The current narrative review is designed to summarize the available literature (both human studies and experimental animal studies) and highlight the gaps in addressing hypertension and CVD risks in offspring of PCOS women or hyperandrogenemic female animal models.

## Introduction

Polycystic ovary syndrome (PCOS) is the most common endocrine disorder affecting women at reproductive age (1). Although the guidelines for diagnosis of PCOS have evolved over time, the currently-used Rotterdam criteria developed by the European Society of Human Reproduction and Embryology/American Society for Reproductive Medicine Rotterdam consensus (ESHRE/ASRM) requires the presence of two out of three features to establish a PCOS diagnosis: oligo- or an-ovulation (OA), clinical and/or biochemical hyperandrogenism (HA), and polycystic ovarian morphology (PCOM) on ultrasound (2).

In addition to being a reproductive and endocrine disorder, PCOS women suffer from metabolic anomalies and obesity, which is their highest priority reason for seeking medical care (3). PCOS women also have increased blood pressure (BP) and increased risk of cardiovascular diseases [CVD] (4), with various involved mechanisms including obesity, sympathetic nervous system activation, renin-angiotensin system (RAS) activation and increased 20-hydroxyeicosatetraenoic acid (20-HETE) in the renal microvasculature [reviewed in (5)]. Given the myriad of signs/symptoms associating PCOS, a lot of questions arise about the health of the offspring. In part they are subjected to the altered *in-utero* environment including maternal hyperandrogenemia. They are also subjected to maternal obesity which has been shown to negatively impact the cardiovascular and metabolic health of the offspring (6–9). The altered maternal environment persists across the lactation period if their mothers are nursing them. There is also a genetic component and the possible transgenerational transmission of PCOS traits (10, 11). Studies have addressed the growth patterns and metabolic and reproductive health of the offspring (12–14). However, very limited studies addressed the risk of developing hypertension in the offspring, which represents a major risk factor for CVD (15), and whether those risks are sex-specific.

## Pregnancy in PCOS women, and associated complications

PCOS women have difficulty getting pregnant and they often require assisted reproduction [e.g. invitro fertilization] (16). PCOS women diagnosed according to the Rotterdam criteria could have one of 4 different phenotypes; full-blown phenotype A (having PCOM, OA and HA), phenotype B (having HA and OA), phenotype C (ovulatory PCOS having HA and PCOM) and phenotype D (non-hyperandrogenic PCOS having OA and PCOM] (17, 18). Decreased fertility in women with PCOS could not be fully explained on the basis of ovulatory dysfunction (19, 20). A comprehensive review utilizing literature from 1970 – 2020 showed that PCOS women exhibit endometrial dysfunction which partly underlies their poor reproductive outcomes. This dysfunction could be due to hyperandrogenemia, insulin resistance, obesity or even chronic inflammation (21). Decreased fertility in PCOS women could also be a result of decreased oocyte competence, which could be influenced by PCOS comorbidities (obesity and insulin resistance) in addition to the differences between the PCOS phenotypes (20).

Studies addressing PCOS pregnancy-associated complications have some discrepancy in their findings. For example, Haakova and colleagues have shown that PCOS is not associated with increased risk of gestational diabetes mellitus (GDM) or pregnancy-induced hypertension (PIH) along with no significance in the gestational weight gain between PCOS and control women (22). On the contrary, the majority of studies found that PCOS women have increased risk of miscarriage, PIH, GDM, pre-eclamptic toxaemia and preterm delivery (23, 24). Homburg and colleagues showed that results of different studies are related to their cohort size, where studies with fairly-small cohorts (n = 22-47) show increased incidence of hypertensive disorders of pregnancy in PCOS, while large-scale studies found no correlation between PCOS and hypertensive disorders of pregnancy [reviewed in (25)]. Another important consideration is the variability of the criteria used for diagnosis of PCOS women enrolled in those studies starting by the NIH 1990 criteria and including the currently-used Rotterdam criteria, with some studies even not mentioning the criteria used. It is also important to think of this discrepancy from the view of the control group, it is possible that the variation in the control group used (whether they are just age-matched healthy women or age-matched and body mass index (BMI)-matched women) is the reason behind the variation seen between the findings of those studies. An extensive review summarizing clinical and pathophysiological features of pregnancy in PCOS concluded that PCOS women have a clinically-significant increased risk (3-4 fold) of pregnancy complications (including PIH, PE, GDM and premature delivery) compared with controls without adjusting for other confounders (including BMI, infertility treatments and others). Women with PCOS still had increased risks of the same pregnancy complications after adjusting for confounders (1.5-2 fold) compared to women without PCOS (26). Unfortunately, the exact pathophysiological mechanism of pregnancy complications in PCOS remains unclear.

The question remains as to whether pregnancy complications are linked to a certain PCOS phenotype. PCOS women diagnosed according to the Rotterdam criteria could have one of 4 different phenotypes; full-blown phenotype A (having PCOM, OA and HA), phenotype B (having HA and OA), phenotype C (ovulatory PCOS having HA and PCOM) and phenotype D (non-hyperandrogenic PCOS having OA and PCOM] (17, 18). Palomba et al., (27), reported increased cumulative rates of adverse obstetric and neonatal outcomes in PCOS women compared to BMI-matched controls. The authors compared the ovulatory PCOS women to the oligo- or an-ovulatory PCOS women and found that the latter group had higher risk of miscarriages, PIH, GDM and operative delivery. Although the risk of pre-eclampsia (PE) was higher in PCOS women than controls, it was similar between ovulatory and oligo- or an-ovulatory women with PCOS (27). The same study categorized women into the 4 Rotterdam phenotypes and showed that the full-blown PCOS (phenotype A) and phenotype B had the highest incidence (93% and 86%, respectively) of adverse neonatal and obstetric outcomes, followed by phenotypes D and C (60% and 22%, respectively) (27). On the other hand, a recent retrospective study that used data extracted from computerized database in France showed that oocyte morphology (essential for fertilization and subsequent fetal development) and percentage of normal oocytes was similar between women with PCOS phenotypes A, C and D (28). In an opinion paper, the author suggested that PCOS women have reduced fertility that is caused by altered oocytes, embryo and endometrial competence, regardless of their ovulatory status (19).

## Offspring birth weight in PCOS; human studies versus experimental animal studies

Studies indicated that PCOS pregnancy could be associated with increased risk of abnormal birth weight in the offspring. The majority of studies showed offspring are born small-for-gestational age (SGA), an indication of intrauterine growth restriction [IUGR] [(24, 29, 30) and reviewed in (23, 25)], and some studies showed offspring are born large-for-gestational age [LGA] (16, 31). The variation could be attributed to the different phenotypes of PCOS, different diagnostic criteria used, different ethnicities of PCOS women or environmental factors surrounding the PCOS women (32) or differences in the pre-pregnancy BMI (33) or maternal diet consumed during pregnancy and lactation. Palomba et al. showed an increased incidence of SGA and LGA and decreased incidence of appropriate-for-gestational age (AGA) babies in women with PCOS compared to controls. The authors showed that only babies from PCOS mothers of phenotypes A and B had significantly higher risk of being born SGA than babies from mothers with phenotypes D and are less likely to be born AGA than babies from mothers with phenotypes D or C, suggesting that the combination of hyperandrogenemia and ovulation disturbance as a key factor for abnormal birth weight in the offspring (27). Interestingly, women with phenotype C and D in this study had a similar percentage of AGA babies as seen in control women of the same study (~ 80 – 86%) (27). Fux-Otta et al. compared pregnancy outcomes from two Latin American populations of women with PCOS and found that offspring from Argentinian PCOS women had higher incidence of SGA babies compared to Chilean PCOS women after adjusting to different maternal factors (32).

Theories behind having SGA babies in PCOS included insulin resistance and insulin-dependent growth dysfunction (25). Another hypothesis is that fetal exposure to excess androgens can induce changes in differentiating tissues, which would also cause the PCOS phenotype to develop in adult life (34). PCOS women with clinical signs of hyperandrogenism have been shown to have decreased endometrial and sub-endometrial blood flow indices (35), which could affect offspring birth weight. Chekir et al. also reported impaired uterine artery perfusion in women with PCOS that correlates with their hyperandrogenemia (36). According to the Barker hypothesis, SGA offspring are at increased risk of metabolic disease, obesity and hypertension as they age (37, 38). LGA offspring are also at increased risk of adverse cardiovascular outcomes with aging (39). Unfortunately, the impact of birth weight on cardiovascular health later in life or the correlation between the maternal PCOS phenotype and the later cardiovascular health in PCOS offspring has not been clearly studied. Gunning and colleagues showed that there was no correlation between maternal androgen levels and offspring BMI or blood pressure at infancy or early childhood (2.5-8 years). Unfortunately, although mothers in this study were diagnosed according to the Rotterdam criteria, the study did not aim at differentiating between the different PCOS phenotypes (33).

On the other hand animal studies have consistently described IUGR in offspring exposed to maternal hyperandrogenemia. It is important to highlight that androgen injection or implantation is the tool utilized by those studies to induce maternal conditions that mimic human PCOS. In sheep, testosterone injections in the dams during early-mid pregnancy (gestational day (GD)30 - GD90; term is 147 days) caused females offspring only (40, 41) or offspring of both sexes (42) to be born with IUGR. In rats, testosterone injections in dams during late pregnancy (GD15 - GD19 of pregnancy; term is 21 days) results in IUGR in female offspring (43) or male offspring (44) or offspring of both sexes (45). Similarly, our studies have shown that maternal exposure to 5 alpha-dihydrotestosterone (DHT) starting prepubertally and continuing throughout pregnancy and lactation results in obesity and insulin resistance in the dams. Importantly, under those conditions of impaired maternal metabolic health and maternal hyperandrogenemia, but no maternal hyperglycemia, offspring of both sexes were still born with IUGR (46–48). Prenatal androgen-induced fetal growth restriction has been attributed to defective transfer of amino acids to the fetus (45), impaired placental function (34) or decreased insulin growth factor availability (42). This could support the suggestion that maternal factors other than hyperandrogenemia (e.g. maternal diet or GDM or other comorbidities) are responsible for having LGA offspring in some PCOS women.

## Do daughters of PCOS women develop hypertension? data from human studies

The pathogenesis behind the development of PCOS is not clear. The origin of PCOS is thought to be multi-factorial and involves an interaction of environmental and genetic factors over the life span. Environmental factors associated with PCOS can be classified into prenatal (intra-uterine environment and fetal developmental programming) or postnatal [diet, obesity, sedentary life style, etc.] (49). Therefore daughters of PCOS women were often studied for their potential to develop PCOS themselves. Some studies show that female offspring of PCOS mothers develop symptoms of PCOS (hyperandrogenemia, cystic ovaries, abnormal menses) with adolescence and young adulthood, and some studies do not, hence whether female PCOS offspring develop PCOS themselves is controversial. For example, some studies found PCOS daughters to have increased insulin (50–52), BMI (53), ovarian volume (52), increased anti-Mullerian hormone (53, 54), menstrual disturbances (54), and increased levels of testosterone pre- and post-pubertal (51, 52, 54). Meanwhile other studies found PCOS daughters to have similar BMI (52, 55) and ovarian volume as controls (56), with no change in insulin sensitivity and glucose tolerance post-puberty (57), and no increase in testosterone in childhood (55) when compared to BMI-matched controls (53, 54). De Leo, et al., suggested a cross-generational relationship between the degree of maternal hyperandrogenism and the development of PCOS in their daughters (58).

Despite those extensive studies on the reproductive and metabolic health of daughters of PCOS women, little is known about their risk for hypertension and CVD. Daughters (4-17 years of age) of PCOS mothers (diagnosed according to the older NIH criteria) had similar BMI, body composition and importantly BP (cuff method) compared to controls at all assessed ages (56). Another study showed that normal weight eumenorrheic daughters of PCOS mothers that have PCOM are still at increased risk of CVD as demonstrated by increased ambulatory BP and decreased plasma nitric oxide (NO) metabolites, along with hyperinsulinemia and hyperglycemia at 24-26 years of age, despite the absence of hyperandrogenemia in those daughters (59).

## Lessons from animal studies in female offspring

PCOS is diagnosed as early as menarche. PCOS women have a 2-3 fold increase in their androgen levels. Studies have shown that women with PCOS maintained a high androgen level throughout pregnancy (60–63) and lactation (64). In addition to this, the majority of PCOS women suffer from a myriad of signs/symptoms that include obesity, insulin resistance and glucose intolerance, all of which could induce reproductive dysfunction and adverse effects on the offspring.

Our group used a rat model of hyperandrogenemia, originally developed by Manneras and colleagues (65), that is induced by subcutaneous implantation of DHT pellets in female Sprague Dawley (SD) rats starting at 4 weeks of age (pre-pubertal) to induce hyperandrogenemia as seen in PCOS women (66). Manneras, et al., showed that the DHT-treated rats exhibited irregular cycles along with PCOM (65). The pellet releases the androgen over a period of 90 days and is replaced every 85 days so hyperandrogenemia is maintained throughout the rat life, as in PCOS, making it suitable for studying different age changes in PCOS (47, 67–69). DHT, unlike testosterone, is a non-aromatizable androgen, therefore does not cause an increase in estradiol levels, neither does it suppress endogenous synthesis of estradiol in rats (66). Upon induction of hyperandrogenemia, rats developed obesity (increased fat mass), insulin resistance, glucose intolerance and an increase in their BP, same as what happens in PCOS women (66). Upon breeding DHT-treated SD females with vendor-supplied SD males, pregnancy occurred in 60% of DHT-treated females, compared to 99% in controls, and offspring are born smaller than control offspring despite the similar liter size, suggesting IUGR as seen in SGA babies from PCOS women (46–48). Thus offspring born to this rat model provide a very realistic tool to assess the CVD risks and their mechanisms in offspring of hyperandrogenemic dams as an experimental model of PCOS.

Female offspring born to DHT-treated females had similar estradiol and testosterone levels compared to control offspring as adults. They did not develop obesity (increased fat mass), had a normal serum lipid profile and normal renal function (indicated by normal proteinuria levels) as adults (46). Surprisingly, female offspring of DHT-treated dams had lower urinary nitrate/nitrite excretion, but remained normotensive. Nitrates/nitrites are metabolites of the vasodilator NO and are used as a measure of endogenous NO levels. We tested their response to exogenous angiotensin (Ang) II after blocking their endogenous RAS by enalapril, an Ang converting enzyme inhibitor, and found that they had a suppressed response to Ang II which could indicate being protected against CVD risks (46). Our results from the female offspring of DHT-treated dams are summarized in **Table 1**. Similar to our studies, using a mouse model injected with DHT during pregnancy (GD16.5 - GD18.5, late pregnancy in mice), female offspring developed cardiac hypertrophy with no change in their BP. The findings in the mouse model were independent of maternal diet and the metabolic profile of the female offspring (70).

**Table 1:** Characteristics of female and male offspring of DHT-treated dams compared to their age- and sex-matched controls.

	Adult male offspring(4-6 months of age)	Adult female offspring(4-6 months of age)	Post-estrous cycling female offspring(16-20 months of age)
Born smaller than controls (intra-uterine growth restriction)	Yes (47, 48)	Yes (46, 47)		
Body weight	Lower (48)	Similar (46)	Similar (46)
Lean mass	Lower (48)	Similar (46)	Similar (46)
Fat mass	Similar (48)	Similar (46)	Similar (46)
Serum total cholesterol	Higher (48)	Similar (46)	Higher (46)
Proteinuria (marker of renal injury)	Higher (48)	Similar (46)	Higher (46)
Urinary nitrates/nitrites (measure of the endogenous vasodilator NO)	Similar (48)	Lower (46)	Similar (46)
Baseline blood pressure	Similar (48)	Similar (46)	Similar (46)
Pressor response to Ang II after blocking endogenous RAS	Exaggerated (48)	Suppressed (46)	Suppressed (46)
Pressor response to Ang II plus salt diet after blocking endogenous RAS	Not tested	Not tested	Suppressed (46)

NO, nitric oxide; Ang, angiotensin; RAS, renin-angiotensin system.

Chinnathambi et al. used a rat model to show that female offspring of dams injected with testosterone (GD15 – GD19, late pregnancy in rats) developed hyperandrogenemia and hypertension as adults. The authors concluded that hypertension in those female offspring was mediated by gonadal testosterone because ovariectomy normalized BP (71). They also showed that female offspring develop a decrease in the levels of NO synthase in their mesenteric arteries, with a decrease in NO-mediated vascular relaxation (72). More and colleagues used a similar model and showed that female offspring develop hypertension as adults, along with an increased contractile response to Ang II in mesenteric arteries due to downregulation of Ang II type-2 receptors (73). King et al. used a sheep model to show that female offspring of dams injected with testosterone (GD30 – GD90, early-mid pregnancy in sheep) also develop mild hypertension as adults. However hypertension is independent of gonadal hormones. It was also independent of plasma aldosterone and catecholamine levels (74). Importantly, testosterone is an aromatizable androgen that is converted to estradiol causing high levels of estradiol during gestation, which raises the question whether the hypertensive effect in the female offspring in those studies is purely androgenic or partly estrogenic.

## Do sons of PCOS develop hypertension, and are there sex differences in CVD risks in offspring of PCOS? data from human studies

Despite the accumulating evidence of the existence of sex differences in the etiology of hypertension and CVD risks (75, 76), studies have not clearly addressed sex differences in the risk of CVD in children born to PCOS mothers. For instance, children of PCOS women, aged 2.5 - 8 years (not separated by sex), had higher aortic pulse pressure, left ventricular internal diameter, and carotid intima-to-media thickness compared to control children (77). The same study pointed out that children of PCOS mothers also had higher triglycerides (TG) and low-density lipoprotein cholesterol (LDL-c) compared to controls (77). Another study that used data from the Western Australia Data linkage system pointed out that PCOS offspring (not separated by sex) are at increased risk of postnatal hospitalizations and were at higher risk of being born with congenital cardiovascular anomalies [1.5% compared with 1.0%, odds ratio 1.37, 95% CI 1.01–1.87] (78), which suggests their increased risk of CVD later in life.

A systematic review and meta-analysis of nine observational studies including offspring of PCOS women from Chile, Netherlands and the US re-analyzed the data with/without stratifying for the sex of the offspring and summarized cardiometabolic health as metabolic sum scores 1 (BMI + systolic BP (SBP) + insulin levels + TG + high-density lipoprotein cholesterol (HDL-c)) and 2 (waist-to-height ratio + SBP + glucose + TG + HDL-c). The authors showed that both metabolic sum scores 1 and 2 were not different between PCOS offspring and control offspring when the data are not stratified for sex; however, with the stratification there was a significant interaction between both sexes, suggesting that sex of the offspring was a significant mediator of cardiometabolic outcomes, when comparing PCOS offspring versus control offspring (13).

A Chilean study recruited sons of women with PCOS (NIH criteria) at 2-3 months of age (infants), 4-7 years of age (children), and 18-30 years of age (adults) to study their metabolic health. The authors showed that sons of PCOS women had increased body weights at all tested ages compared to controls, and had insulin resistance with hyperinsulinemia as adults (79). Later on, the same group showed that sons of women with PCOS (NIH criteria) at 7-18 years of age had hypercholesterolemia and increased LDL-c compared to controls (80). Unfortunately, neither study addressed BP in PCOS sons. Thus it remains unclear whether sons of women with PCOS are at increased risk of developing hypertension or CVD as adults or not.

## Lessons from animal studies in male offspring

Using our DHT-treated rat model explained earlier, adult male offspring had a decrease in their body weights that is mediated by a decrease in their lean mass. They also developed hypercholesterolemia and increased urinary protein excretion (marker of renal injury). Importantly, although those males remained normotensive at baseline, they had an exaggerated pressor response to chronic Ang II after blocking their endogenous RAS with enalapril (**Table 1**) (48). This could suggest an increased risk of hypertension and CVD with aging in male offspring of hyperandrogenemic dams.

Chinnathambi, et al., showed that although both male and female offspring of testosterone-treated dams (rat model) develop hypertension as adults, the increase in BP in prenatal testosterone-exposed adult males was more pronounced than in females (71). Unlike female offspring, male offspring of testosterone-treated dams developed a decrease in endothelium-derived hyperpolarizing factor-mediated relaxation of mesenteric rings (72). Injecting testosterone directly into the flanks of male fetuses (GD62 - GD82, post-sexual differentiation in sheep fetus) resulted in dyslipidemia and altered metabolic health (hyperinsulinemia with normal testosterone levels) (81), but the authors did not address BP.

## What happens to BP in offspring of PCOS with aging?

Currently-used criteria for diagnosis of PCOS (Rotterdam criteria) have been in place since 2003/4, which makes offspring of the population of women diagnosed according to this diagnosis paradigm still in their late teens (2). Older criteria (NIH) were in place in 1990, so offspring of this population are now in their third decade of life. Therefore, human studies so far have not addressed the question of whether maternal hyperandrogenemia as seen in PCOS would impact cardiovascular health of the offspring as they age. In fact, studies addressing the impact of aging on the cardiovascular health of PCOS women themselves are still lacking. It is well-accepted that males are at increased risk of hypertension and CVD when they are young, compared to age-matched women [reviewed in (82)]. According to the National Health and Nutrition Examination Survey conducted on 9,623 participants, the prevalence of stage 2 hypertension (SBP/DBP >/= 140/90 mmHg) among women older than 75 years was 78% compared to 71% in men. Also the prevalence of stage 1 hypertension (SBP/DBP >/= 130/80 mmHg) among women older than 75 years was 85% compared to 79% in men (83). The question still remains as to whether male and female offspring of PCOS mothers will carry exaggerated risks of CVD with aging or not.

Using the DHT-treated female rats, we have shown that female offspring at 16-18 months of age (post-estrous cycling) remained normotensive, despite decreased renal function (higher proteinuria). They had lower heart rates compared to the adult female offspring of DHT-treated females, suggesting further damage to the heart function with further aging. Post-estrous cycling female offspring of DHT-treated dams also had similar fat mass and body weight, but higher serum total cholesterol compared to age-matched controls. We challenged them with exogenous angiotensin II after blocking their endogenous RAS with enalapril to determine their pressor response. Post-estrous cycling female offspring of DHT-treated dams had a suppressed pressor response to Ang II and Ang II plus 4% salt diet, which could be partly due to increased intrarenal Ang 1-7 (vasodilator arm of RAS). Our future studies will aim at addressing CVD risk in the female offspring with further aging, and in male offspring (46).

## Impact of life style, diet or different medications received during PCOS pregnancy on offspring cardiovascular health

Another great gap in our knowledge is the impact of different factors including the PCOS mother`s life style and medications on the offspring cardiovascular health and their risk of developing hypertension. For example, some studies pointed out an increased prevalence of PCOS in western diet (WD)-consuming females (84, 85). Szczuko, et al., even suggested that the improper diet is the main reason behind metabolic abnormalities in PCOS (86). However, studies addressing the impact of maternal diet in PCOS on offspring cardiovascular health or risk of hypertension are lacking. Bishop and colleagues have shown that in Rhesus Macaques, testosterone-treated females require increased time to achieve pregnancy; however, WD-fed Macaques had decreased numbers of pregnancies and overall fertility to 70%. Importantly, testosterone-treatment plus WD consumption simultaneously decreased the number of viable fetuses compared to either testosterone or WD alone (87). Testosterone plus WD also promoted an increase in fasting blood glucose level and impairment of glucose tolerance during pregnancy (87), which could suggest further metabolic derangements in the offspring as they age. However, using a mouse model, Risal, et al., showed that prenatal androgen exposure, but not maternal WD consumption, causes transgenerational reproductive and metabolic dysfunction in female offspring (11). Neither addressed the cardiovascular health or BP of offspring with aging.

Another example is the use of metformin by pregnant PCOS women and its effect on their offspring BP as they go into adulthood. Various studies have addressed the role of metformin in reducing the incidence of miscarriage and GDM in pregnant PCOS [(88) and reviewed in (89, 90)]. Others found that metformin treatment during the whole pregnancy in PCOS could reduce the risk of IUGR in offspring [from 17%-22% in groups untreated with metformin or treated for only certain period during their pregnancies to 2% in group treated with metformin across the whole pregnancy] (88). On the contrary, a follow-up study on two randomized controlled trials showed that metformin-exposed children had higher BMI and increased prevalence of overweight/obesity at 4 years of age (91). Using a rat model of PCOS, Xie, et al., showed that metformin treatment during pregnancy reduces the risks of insulin resistance and obesity in female offspring (92).

Torstein and colleagues performed a follow-up study on a randomized clinical trial to include children of PCOS women (~ half of them received metformin during pregnancy and the other half were placebo-treated) with an average age of 8 years. In both metformin and placebo groups, BP in the offspring was within the normal range for the gender and age studied. However, SBP was higher in the metformin group (106 mmHg vs. 101 mmHg) with a borderline significance of p = 0.05. Meanwhile, DBP was not different between the groups. This study has a very limited number of participants with the majority of the metformin group being boys and the majority of the placebo group being girls, and data were not stratified according to sex most likely because of the limited number of participants (93).

## Conclusion

**Figure 1:**
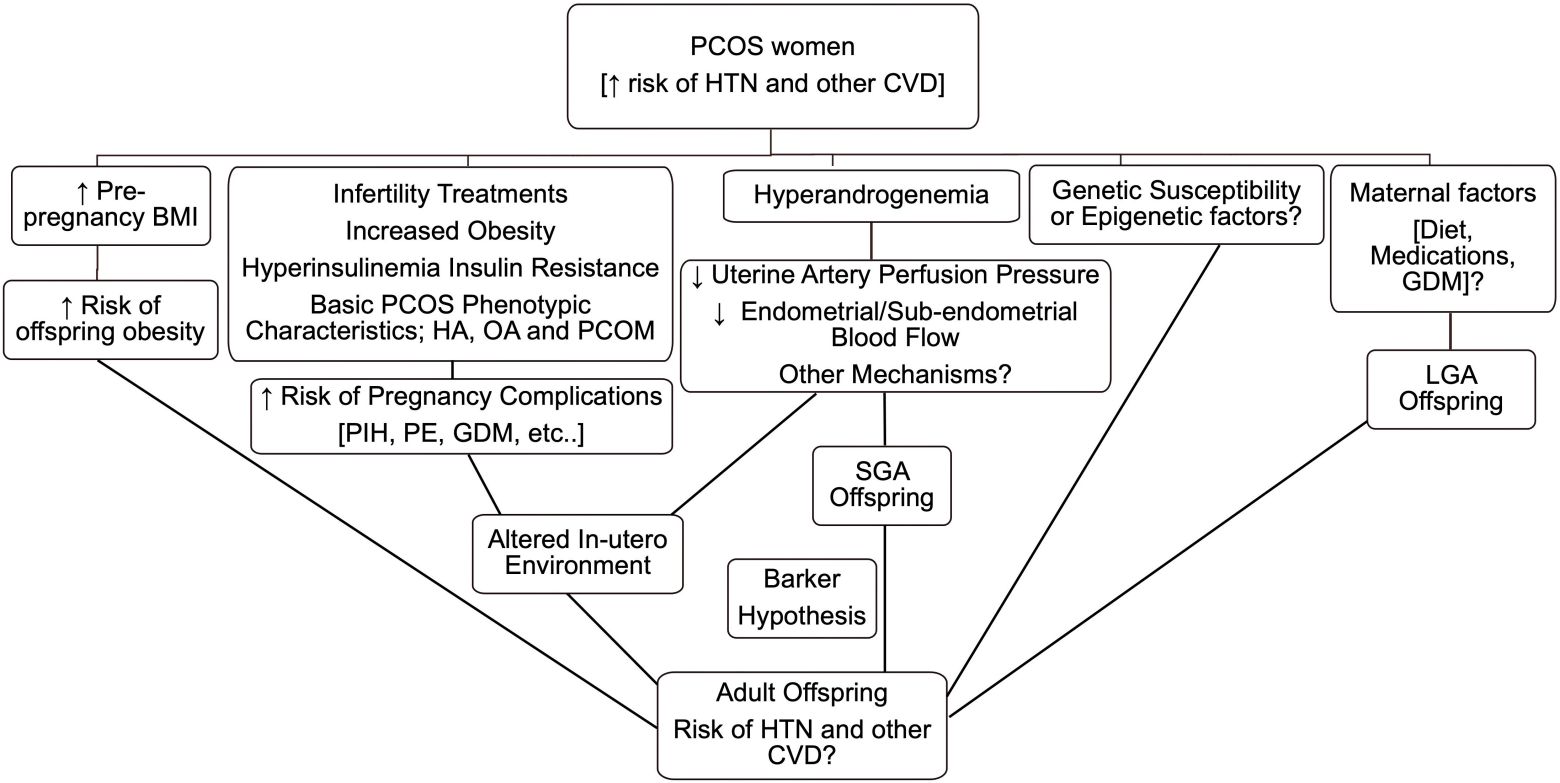
Rationale and possible mechanisms behind increased risk of hypertension (HTN) and other cardiovascular diseases (CVD) in offspring of polycystic ovary syndrome (PCOS) women. BMI, body mass index; HA, hyperandrogenism; OA, oligo- or an-ovulation; PCOM, polycystic ovarian morphology; GDM, gestational diabetes mellitus; PIH, pregnancy-induced hypertension; PE, pre-eclampsia; LGA, large-for-gestational age; SGA, small-for-gestational age.

Based on their abnormal birth weight, exposure to altered maternal environment both *in-utero* and during lactation and possible trans-generational transmission of some PCOS traits, the risk of hypertension and CVD in offspring of PCOS may be strongly predicted as shown in **Figure 1**. However, studies have not yet clearly addressed those risks in either adult or aging offspring. Sex differences are strongly suggested and should be examined further. Animal studies paying attention to duration and timing of exposure of dams to hyperandrogenemia and the associated metabolic abnormalities could provide a useful tool for determining the impact of maternal hyperandrogenemia plus maternal obesity (as seen in obese PCOS) on offspring cardiovascular health as adults, and even more importantly with aging.

## Author contributions

NS conceptualized and designed the study, performed the literature search, drafted and revised the review article.

## Funding

Research reported in this publication was supported by the National Institute of General Medical Sciences of the National Institutes of Health under Award Number P20GM121334 (N.M.S). The content is solely the responsibility of the author and does not necessarily represent the official views of the National Institutes of Health. This work was also supported by the American Heart Association Career Development Award Number 938320 (N.M.S.).

## Conflict of interest

The author declares that the research was conducted in the absence of any commercial or financial relationships that could be construed as a potential conflict of interest.

## Publisher’s note

All claims expressed in this article are solely those of the authors and do not necessarily represent those of their affiliated organizations, or those of the publisher, the editors and the reviewers. Any product that may be evaluated in this article, or claim that may be made by its manufacturer, is not guaranteed or endorsed by the publisher.
